# Calcium Silicate-Based Sealer Dentinal Tubule Penetration—A Systematic Review of In Vitro Studies

**DOI:** 10.3390/ma16072734

**Published:** 2023-03-29

**Authors:** Israa Ashkar, José Luis Sanz, Leopoldo Forner, María Melo

**Affiliations:** Department of Stomatology, Faculty of Medicine and Dentistry, Universitat de València, 46010 Valencia, Spain

**Keywords:** bioceramic, calcium silicate, endodontics, in vitro, penetration, sealer, systematic review

## Abstract

The aim of this systematic review was to perform a qualitative synthesis of in vitro studies which evaluate and compare the penetration of calcium silicate-based sealers into dentinal tubules. A systematic advanced search was performed in Scopus, Embase, Medline (via PubMed), Web of Science, and Cochrane databases on the 1 December 2022. In vitro studies that compared the tubular penetration of at least two calcium silicate-based sealers in extracted human teeth were eligible. PRILE 2021 guidelines were used for the assessment of the risk of bias included studies. The search identified a total of 680 preliminary records, among which 12 studies were eligible for review. The most used methodology to evaluate sealer penetration was the use of a fluorochrome together with the tested sealers and the analysis of their penetration under confocal laser microscopy. Regarding the results of the included studies, calcium silicate-based sealers exhibit a favorable dentinal tubule penetration. Tubular penetration, however, can be affected by factors such as the irrigation protocol, sealer activation, the filling method used, and root canal morphology. EndoSequence BC Sealer showed the highest sealer penetration among the tested sealers. The influence of different fluorochromes on the results of dentinal tubule penetration studies should also be further explored. The in vitro nature of the included studies limits the applicability of the results into the clinical setting. Prospero registration: CRD42022383896

## 1. Introduction

During root canal treatment, the root canal system is disinfected in a chemical-mechanical manner to reduce its microorganism load and remove any tissue debris within it. It is followed by a three-dimensional filling which provides a hermetic seal from the coronal orifice of the canal to the apical foramen [1]. Establishing a well obturated root canal system is crucial to prevent the coronal leakage of microorganisms and to provide a biocompatible medium that allows the repair of any existing periapical lesions or to prevent the development of new lesions [2].

From a histological perspective, a correct three-dimensional seal is majorly based on the penetration of the materials placed inside the root canal into the dentinal tubules. A higher penetration increases the contact surface between the dentin substrate and the filling material, granting a greater sealing ability which can potentially prevent the penetration of new microorganisms and trap any remaining ones [3]. The reduction in the microorganism load plays a crucial role in clinical success rate of endodontic treatment and the healing process of existing periapical lesions [4]. Thus, parameters such as the percentage, depth, and area [5,6] of dentinal tubule penetration, along with antimicrobial properties [7], are relevant when assessing the adequacy of endodontic sealers for clinical use.

Various techniques have been proposed for root canal filling, such as cold lateral compaction, single cone technique, Tagger’s hybrid technique, carrier-based obturation, and continuous wave. These techniques are based on the combination of gutta-percha and various compositions of root canal sealers [8], such as zinc oxide eugenol, glass ionomer, calcium hydroxide, silicone, epoxy resin, and calcium silicate cement-based sealers [9].

The use of calcium silicate-based cements in the field of endodontics began with the introduction of Mineral Trioxide Aggregate (MTA) by Dr. Torabinejad [10]. MTA is a Portland cement-based formulation which was initially developed for root perforation repair, retrograde filling after apicoectomy, and pulp capping [11]. Today, new formulations of cements which are no longer based on Portland cement, but on tricalcium silicate or dicalcium silicates, are gaining popularity as root canal sealers due to their antimicrobial properties, biocompatibility [12], alkaline pH, and bioactivity (i.e., their ability to form hydroxyapatite on their surface and form a mineral attachment to the dentin substrate) [13,14]. Their physicochemical and biological properties are comparable to those exhibited by the established epoxy resin-based sealers [15], making them a viable alternative for root canal sealing. The general composition of calcium silicate-based sealers includes varying percentages of tricalcium or dicalcium silicates, a radiopacifying agent, a mixing liquid (in the case of powder-liquid compositions), and additives [14].

The ability of sealers to penetrate dentinal tubules is determined by essential physicochemical properties such as the sealer’s flowability, solubility, and setting time [16]. Sealer flow is an essential property that allows the sealer to fill difficult-to-access and complex areas, such as the narrow irregularities of dentin and accessory canals [14]. Factors that influence the flow rate of sealers include particle size, temperature, and mixing time. According to the International Standards Organization (ISO) 6876 standard for root canal sealing materials, the solubility of a sealer shall not exceed 3% mass fraction after immersion in water for 24 h [17]. This is because a highly soluble sealer can cause gaps along the dentin/sealer/gutta-percha interface that might offer a pathway for bacteria and their byproducts into periapical tissues [18,19]. Alternatively, longer setting time allows the penetration of the sealer into the root canal morphology more readily after its placement [20].

Calcium silicate-based sealers are a group of biomaterials which base their setting reaction on water and hydroscopic inorganic components (hydraulic setting). They are available either in a powder/liquid format or pre-mixed form [21,22]. The pre-mixed format requires an external source of water (from the dentinal tubules) to carry out their setting reaction, while the powder-liquid format will start its setting reaction once both components are mixed i.e., before their application in the root canal [23].

The analysis of endodontic sealer penetration is often assessed in laboratory studies by sectioning filled extracted tooth perpendicular to the vertical axis of the root canal [24,25,26] and then evaluating the penetration of the sealer using scanning electron microscopy (SEM) or confocal laser scanning microscopy (CLSM). The latter provides detailed information about the distribution of sealers inside dentinal tubules at relatively low magnification using fluorescent-marked sealers [27].

Over the past decade, various in vitro studies were carried out to evaluate and compare sealer penetration, particularly by comparing calcium silicate-based sealers and epoxy resin-based sealers together [3,28,29]. According to a previous systematic review [30], the latter exhibit a superior sealer penetration over calcium silicate-based sealers. Nevertheless, calcium silicate-based sealers are still in uprising clinical use due to their favorable properties [31]. Consequently, a high number of in vitro studies compare the dentinal tubule penetration of different calcium silicate-based sealer compositions.

Two recent studies [32,33] have critically assessed and criticized the commonly used methodology for the assessment of sealer penetration from previously published in vitro studies. Nevertheless, there is still a high number of studies which have used such methodology and no effort has been made to perform a qualitative synthesis of their results.

Accordingly, the aim of this systematic review is to present a qualitative synthesis of available evidence on calcium silicate-based sealer penetration from a critical perspective, considering the recent controversy.

## 2. Materials and Methods

### 2.1. Protocol and Registration

The present work followed the guidelines recommended by PRISMA 2020 Statement (Preferred Reporting Items for Systematic Reviews and Meta-analysis) [34]. The systematic review protocol was previously registered in the Prospective Register of Systematic Reviews (PROSPERO), University of York, with the registration number CRD42022383896.

### 2.2. Eligibility Criteria

In vitro studies that compared the dentinal penetration of at least two calcium silicate-based sealers in extracted human teeth were eligible. The inclusion criteria were based on the PICOS framework [35] as follows:

Population (P): extracted teeth; intervention (I): root canal treatment with calcium silicate sealer-based filling; comparison/control (C): calcium silicate-based sealers; outcome (O): dentinal tubule penetration of sealers in terms of depth, percentage, and area of penetration of the sealer around the canal perimeter; and study design (S): in vitro.

### 2.3. Information Sources and Search Strategy

The search process, study selection, methodology and outcome data extraction, and quality assessment were carried out by two examiners (I.A and M.M). A third examiner was consulted in the event of any doubt (L.F.). A systematic advanced electronic search was performed in Scoups, Embase, Medline (via PubMed), Web of Science, and Cochrane databases on 1 December 2022, without any date or language restrictions. The following terms were used “bioceramic”, “silicate”, “sealer”, “endod*”, “root canal”, “penetrat*”, and “adhesion”. Boolean operators “AND” and “OR” were used to annex the terms and develop the search strategy. The full search strategy is presented in Table 1. Additionally, the references of the included studies were screened for potentially eligible studies that did not appear in the preliminary database search.

### 2.4. Study Screening and Selection Process

The records resulting from the search strategy were exported from each database into Mendeley reference manager software (Elsevier, Amsterdam, The Netherlands) and duplicate records were discarded manually. Subsequently, an initial screening of the titles and abstracts of the resulting records was performed. Then, the full texts of the studies which met the inclusion criteria in the first screening were retrieved and an additional assessment was performed to confirm their eligibility.

### 2.5. Data Collection Process and Data Items

Data extraction was subdivided on variables on study characteristics, methodology, and results. Regarding the study characteristics, the author and year of publication were extracted. Regarding the study methodology, the following variables were extracted: extracted teeth, teeth storage after extraction, distance from the endodontic instrument to the apical foramen, instrumentation system, irrigation sequence, sealers used, dyes used, teeth storage after filling, and teeth sectioning distance from apex. Lastly, regarding the study results, both the outcome measured and the method to assess the outcome were extracted.

### 2.6. Study Risk of Bias Assessment

The “PRILE 2021 guidelines for reporting laboratory studies in Endodontology” [36] were used for the evaluation of inner methodological quality assessment (risk of bias) of the included studies resulting from the selection process. For each of the 40 parameters considered in the quality assessment tool, studies were individually assessed for fulfilment/non-fulfilment and the percentage of complied items was subsequently calculated (number of complied items/total number of items × 100).

## 3. Results

### 3.1. Study Selection

**Figure 1:**
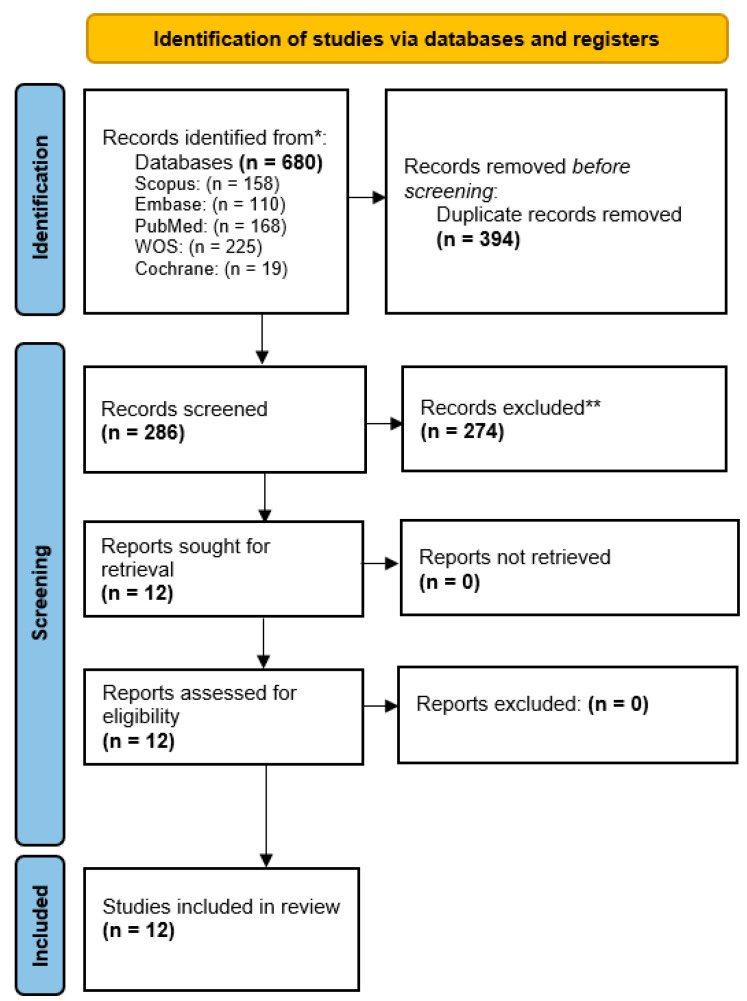
Systematic flow chart representing the study search and selection process. Based on the PRISMA 2020 flow diagram [34]. * Records were retrieved from electronic databases. Repositories and registries were not used as a source. ** Records were excluded after title and abstract screening if one or more of the inclusion criteria were not met.

The search identified a total of 680 preliminary results, where 158 articles were found in Scopus, 110 in Embase, 168 in Medline (via Pubmed), 225 in Web of Science, and 19 in Cochrane. Duplicates were manually discarded using Mendeley reference management software, resulting in 286 records. From there, 274 records were excluded upon screening the title and abstract. The 12 resulting articles were evaluated by reading their full text, and all 12 of them were considered as eligible for qualitative synthesis after full-text evaluation: [37,38,39,40,41,42,43,44,45,46,47,48] (Figure 1). No additional studies were found upon the manual reference searching of eligible studies.

### 3.2. Study Methodology

#### 3.2.1. Studied Materials

The list of the commercially available calcium silicate-based sealers assessed by the included studies, along with their manufacturers, and compositions are presented in Table 2.

#### 3.2.2. Sample Characteristics, Instrumentation, and Irrigation Sequence

The summary of the methodology of the included studies in terms of sample characteristics, instrumentation and irrigation sequence used are showed in Table 3. From the study sample (*n* = 12), all studies used extracted human teeth with single canals whether incisors, canines, or premolars. The general inclusion criteria in the studies included intact, fully formed root and apices without root resorptions, carious lesions, cracks, fractures, or previous root canal treatments. Eight studies reported the methodology used to store the teeth after extraction, which varied from: distilled water, formalin, 0.9% NaCl solution, saline, thymol, or 100% humid environment. Four studies did not report the storage medium after extraction. All studies used a K-file to establish patency and the working length where the distance from the tip of the file to the apical foramen ranged between 0–1 mm. Instrumentation and irrigation sequence varied between all studies. Particularly, three of the included studies used different final irrigation protocols to assess their effect on the penetration of the sealers.

#### 3.2.3. Study Groups and Outcome Measurement

The summary of the methodology of the included studies in terms of the study groups and outcome measurement are showed in Table 4. Sample size per group was varied, ranging from 8 to 39 roots per group. One of the included studies [37] ultrasonically activated the sealers inside the root canals. All studies mixed the sealers with a fluorescent dye to allow visualization of the sealer penetration in the dental canals except one study [42] which did not mix the sealer with any dye. After root canal filling, all samples in all studies were stored in 100% humidity for 3–14 days and were later sectioned horizontally in different distances away from the apex to obtain samples from the apical, middle, and coronal thirds of the root. The sealer penetration assessment method was observed by using Confocal laser scanning Microscope (CLSM) by 10 studies, while Alim et al. [38] and Marissa et al. [42] used Cytation 5 Cell Imaging Multimode Reader and Gen5 software, and scanning electron microscopy (SEM), respectively.

**Figure 2:**
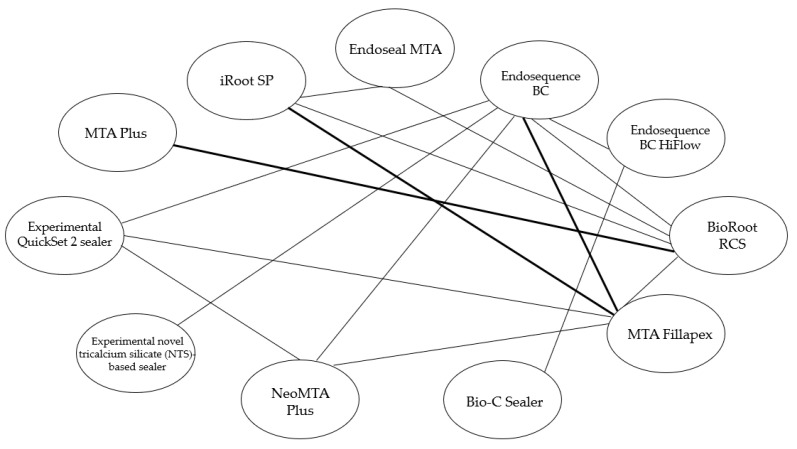
Schematic representation of the number of comparisons between calcium silicate-based sealers among the included studies. Line weight key: Thin- Sealers compared one time, Thick-Sealers compared twice.

The different comparisons made between calcium silicate-based sealers among the included studies are depicted in Figure 2. It can be highlighted that EndoSequence BC sealer was the most used sealer, compared with six different sealers, followed by BioRoot RCS and MTA Fillapex which were compared with four different sealers, and iRoot SP and NeoMTA Plus which were compared with three different sealers. However, most comparisons were only made once in separate studies, except for “EndoSequence BC and MTA Fillapex”, “BioRoot RCS and MTA Plus”, and “MTA Fillapex and iRoot SP” which were compared twice in separate studies.

### 3.3. Study Results

#### 3.3.1. Sealer Tubular Penetration Depth

The qualitative significant differences reported by the included studies in terms of sealer tubular penetration depth are presented in Table 5. The quantitative data on sealer tubular penetration depth are presented in Supplementary Tables S1 and S2.

In all the included studies, the sealer penetration into the dentinal tubules was lower apically and increased coronally. Endodecuence BC sealer was the most compared with other sealers. It showed a significantly higher penetration depth than experimental QuickSet2 sealer and BioRoot RCS in two studies [40,47]; respectively. Alternatively, MTA Fillapex showed a significantly higher sealer penetration depth than experimental QuickSet2 in one study [47], and iRoot SP and BioRoot RCS in another study [42].

Three of the included studies [39,41,47] filled the canals with sealers but used different obturation methods to evaluate whether the filling technique had an effect on tubular penetration depth or not. Two of these studies reported significantly higher calcium silicate-based sealer penetration when using the warm vertical technique in comparison with the single cone technique when using EndoSequence HiFlow BC sealer and Bio-C sealer [39] and when using MTA Fillapex and EndoSequence BC Sealer [47].

Two of the included studies [38,45] used different final irrigation protocols to assess their effect on the sealer penetration depth, Alim et al. [38] reported that using maleic acid (MA) as a final irrigation had a better effect on the outcome of MTA Flillapex and EndoSequence apically, allowing deeper sealer penetration into the dentinal tubules in comparison with EDTA and HEBP (etidronic acid). Aktemur et al. [45] reported that though the smear layer did not affect the penetration depth of root canal sealers, the penetration depth of MTA Plus was significantly higher compared to BioRoot RCS when the smear layer was preserved, while BioRoot RCS showed the lowest penetration depth when the smear layer was removed with 17% EDTA.

#### 3.3.2. Sealer Penetration Percentage and Area

Five of the included studies [37,40,41,45,47] evaluated the penetrated sealer percentage into dentinal tubules and only two found significant differences between the groups. Mahrani et al. [37] found that Endoseal MTA^®^ with sealer ultrasonic activation exhibited a higher penetration percentage than EndoSeal when placed without sealer activation. However, iRoot SP with and without activation in the same study, showed good but no significant differences. Muedra et al. [40] also observed significant difference between the groups as EndoSequence BC had higher penetration percentage the BioRoot RCS in all three thirds of the roots.

The sealer penetration area was only assessed by Akcay et al. [48], where the overall values of iRoot SP exhibited significantly higher sealer penetration area than MTA Fillapex, regardless of the final irrigation techniques used in the study.

### 3.4. Quality Assessment

The results from the quality assessment using the PRILE 2021 guidelines for reporting laboratory studies in Endodontology [36] are presented in Supplementary Table S3. The mean compliance of the included studies was 77% with a maximum score of 94% and a minimum score of 61%. All in vitro studies included in this review provided the area/field of interest in the tile (item 1b) as well as at least two keywords related to the subject of the investigation (item 2a). Within the abstract, all studies presented clear objectives of the investigation (item 3b) and the main conclusions of the study (item 3e). All studies also managed to provide a background summary of the scientific investigation with relevant information (item 4a) and the aim or hypothesis of their investigation (item 4b) in the introduction.

Furthermore, in the Materials and methods section, item 5d, which indicated providing sufficient information about the methods/materials/supplies/samples/specimens/instruments used in the study, and item 5e which indicated a defined and reliable use of the study’s categories were also fulfilled by all the included studies. However, item 5b, which implies the use of applicable procedures when harvesting cells for research respecting all the legal, ethical, and welfare rights of human subjects was only fulfilled by two studies. Likewise, all included studies failed to fulfil item (6b) in the results section except for one study that reported information on the loss of samples during experimentation. Regarding the discussion section, two items were fulfilled by all the included studies, since they all described the relevant literature and status of the hypothesis (7a) and the true significance of the investigation (7b). Both items related to the conclusion section were also all fulfilled by all the studies (8a,8b). Lastly, items 11a,b,e and h regarding to the quality of images were also fulfilled by every included study. Items 5f–i were deemed as non-applicable to the included studies.

## 4. Discussion

It was formerly demonstrated in a previous systematic review [30] that calcium silicate-based sealers showed inferior dentinal tubular penetration than epoxy resin-based sealers. The latter are considered the “gold standard” sealers in clinical practice and the reference materials in both laboratory and clinical research [55] due to their excellent physical properties, including low solubility, high flow rates, and low-volume polymerization shrinkage [56]. However, the use of calcium silicate-based sealers in clinical practice is becoming increasingly popular, due to their biocompatibility [57], antimicrobial substantivity [58], and bioactivity [59]. The last property results in the formation of a superficial hydroxyapatite-like crystalline structure which improves material-to-dentin sealing [60]. Taking this into account, the aim of this systematic review was to synthesize the evidence in the available literature on the tubular penetration of calcium silicate-based sealers. To the authors’ knowledge, this systematic review is the first to compare this parameter within the material subgroup of calcium silicate-based sealers.

### 4.1. On the Methodology of the Included Studies

As mentioned previously, three different observation methods were used among the included studies. Among them, CLSM is especially useful because it can clearly visualize the infiltration of sealer tags into dentinal tubules with few artifacts [61,62]. In addition, it does not promote specimen dehydration [63] and can provide a detailed view of interfacial adaptation and the distribution of sealers using fluorescence [64]. This is because it has a high contrast which allows an appropriate analysis of the sealer in the dentinal tubule, even from thick specimens without the previous sample preparation [65,66].

On the other hand, SEM requires a prior sample preparation, including sample dehydration, demineralization, polishing, and observation under high vacuum. This may result in the production of artifacts, leading to artificial gaps which may hinder the analysis of the sealing interface [67,68]. Interestingly, Cytation 5 Cell Imaging Multimode Reader is a new system that has not been used in other sealer penetration studies. This system can perform imaging considerably faster and in an easier manner than CLSM and display up to 96 samples at once at specified wavelengths [38]. Hence, the use of this system should be considered in future dental material penetration studies, especially in studies involving large numbers of samples.

All included studies that used CLSM labelled the sealer with rhodamine B fluorescence to identify the sealer within the dentinal tubules. Of the dye, 0.1% was mixed with the sealers in most of the studies, whereas one of the studies used 0.01% [38]. Different methods were reported among the studies with regards to mixing the sealers with the rhodamine B dye. For example, an endodontic explorer can be used to mix a trace amount of dye with the sealer [41,47]. Alternatively, 0.002 g of Rhodamine B can be added to 1 g of endodontic sealer [40]. Lastly, another study proposed manually mixing 10 parts of sealer with 1 part of dye powder [46]. Rhodamine B is known to have a powerful affinity for moisture and less affinity for calcium in the sealer composition. Therefore, it has been suggested that it can separate from or leach out of its mixture with the sealer, trace even small degrees of moisture in dentin, emit fluorescence independent of the sealer, and show deeper penetration into the dentinal tubules; resulting in inaccurate results [69]. However, Patel et al. conducted a pilot study prior to their investigation on the penetration of two sealers depending on the presence or absence of rhodamine B dye. They found that the penetration results were similar regardless of the presence or absence of rhodamine B dye. Consequently, the possibility of false results due to leaching of rhodamine B from the sealers was according to the authors, excluded [70]. Taking this into account, it was proposed in the past that if a small amount of this dye is used (less than 0.2%), rhodamine B provides a correct identification of the sealer and has no effect on its physical properties [71,72]. Nevertheless, the recent study conducted by Donnermeyer et al. [33] states that labelling the sealers with rhodamine B is an inadequate method with which to evaluate sealer penetration depth into dentinal tubules, since the staining does not fixate or bind to the sealers. In this way, the penetration depth into the dentinal tubules is can appear as an overestimation.

For this reason, the use of Fluo-3 dye as a fluorophore was suggested by a recent study [73] to evaluate dentinal tubule penetration of calcium silicate-based sealers. Fluo-3 is a non-fluorescent compound. However, its fluorescence significantly increases after binding to calcium. In this manner, the calcium present in the calcium silicate-based sealers binds to Fluo-3. Consequently, the observed fluorescence comes from the sealers [69,74]. Additionally, Fluo-3 is not able to detect calcium ions from the dental structure. Therefore, the obtained results are based only on the calcium from the sealer composition [69]. To support this statement, previous studies that used Fluo-3 dye [69,73] to evaluate the sealer penetration depth of calcium silicate-based sealers showed substantially inferior sealer penetration compared to values from studies using rhodamine B dye. Altogether, available evidence on the characteristics of Fluo-3 indicate that it may serve as a suitable alternative to rhodamine B for this type of studies, though more studies are needed to prove this methodology.

### 4.2. On the Results of the Included Studies

Regarding the tested materials, EndoSequence BC sealer and MTA Fillapex were the most used sealers. EndoSequence BC Sealer showed excellent sealer penetration in all the studies, and it penetrated significantly deeper than BioRoot RCS [40]. The higher sealer penetration of EndoSequence BC sealer compared to other calcium silicate-based sealers could be attributed to the size of the sealer particles (<1 um) [43] and to the fact that this sealer comes in a premixed form. According to Muedra et al. [40] this ready-to-use form may exhibit higher sealer penetration than powder/liquid sealers, since even when following the manufacturers’ instructions for the preparation of sealers, small variations in the dosage during the mixing process may occur.

MTA Fillapex was compared with EndoSequence BC sealer in two separate studies [38,47]. In both studies, EndoSequence BC showed better but not significantly different tubular penetration compared to MTA Fillapex. This could be associated to the fact that MTA Fillapex shrinks up to 0.7% during setting, whereas the EndoSequence BC Sealer in fact slightly expands (<0.1%) during setting [75].

iRoot SP and MTA Fillapex were also compared in two different studies. Interestingly, they exhibited different results in both studies, even though both were used with the same filling method (single cone technique). In the first study, MTA Fillapex showed a significantly higher sealer penetration depth than both iRoot SP and BioRoot™ RCS 5 mm away from apex [42]. The authors suggested that this could be attributed to the flow rate of MTA Fillapex, which is more fluid than the other two sealers [19]. The presence of resin in the composition of MTA Fillapex may also have an effect of this outcome. As described in a previous laboratory study [76], calcium silicate-based sealers which contained a mixture of tricalcium silicate and resin exhibited a higher flow than those containing calcium phosphate silicate (IRoot^®^ SP, BioMed Central, London, UK) and pure tricalcium silicate (BioRoot™ RCS). In the second study, iRoot SP showed a higher penetration depth than MTA Fillapex. This may be attributed to iRoot SP’s small particle size [77], and to the fact that it exhibits minimal or no shrinkage during its setting phase [78], or even to its 0.2% expansion during the setting period [79]. The differences between the outcomes in both included studies regarding these sealers may be due to the different observation methods used (CLSM with dye and SEM without dye, respectively).

The two other sealers that were compared in two separate studies were BioRoot RCS with MTA Plus. The two sealers showed no significant differences in tubular penetration in one of the studies [46], whereas BioRoot RCS showed a lower sealer penetration depth than MTA plus in the other [45]. Parallelly, BioRoot RCS exhibited less sealer penetration compared to other calcium silicate-based sealers such as MTA Fillapex, iRoot SP, and EndoSequence BC sealer in other studies [40,42]. It should be highlighted that BioRoot RCS demonstrated a higher penetration depth compared to Endoseal sealer in the middle and coronal third in an included study conducted by Kim et al. [44].

### 4.3. Factors which Influence Dentinal Tubule Penetration

Various included studies assessed tubular penetration in terms of the irrigation sequence, irrigant and/or sealer activation, and root canal filling method. Three of the included studies [39,41,47] filled the canals with the same sealer but with two different filling methods (single cone and warm vertical compaction) to evaluate whether the filling technique influenced sealer penetration. One of the studies [39] found that EndoSequence BC sealer HiFlow and Bio-C sealer penetrated significantly deeper with warm vertical compaction technique compared with the single cone technique. Likewise, another study [47] found that MTA Fillapex and EndoSequence BC Sealer showed the same trend.

These outcomes contrast with the results of the study conducted by Reyonolds et al. [41], who found no significant difference in sealer penetration regarding the filling method. The same occurred in another study [69] in which the authors asserted that the pressure derived from warm vertical compaction technique did not enhance the penetration depths of the calcium silicate-based sealer. In fact, the use of calcium silicate-based sealers is typically recommended with single cone technique, since the heat may affect or deteriorate their physical properties by decreasing their bond strength, shortening their setting time, and reducing their flow rate [80]. However, a previous study found that calcium silicate-based sealers were not actually influenced by heat [81] and another study described that lateral canals are more easily filled with warm vertical compaction technique [82]. Altogether, the dissimilarities among studies in the calcium silicate-based sealer penetration depending on the filling method emphasizes the need for further investigation in this regard.

Various studied evaluated the influence of several factors on calcium silicate-based sealer penetration, including the irrigation protocol and sealer activation. Three included studies used different final irrigation protocols to evaluate their effect on sealer penetration. Akcay [48] reported that the use of Phophoton-induced-photoacoustic streaming activation (PIPS) or passive ultrasonic irrigation (PUI) in the final irrigation allowed iRoot SP, MTA Fillapex, and GuttaFlow Bioseal to penetrate significantly deeper into the dentinal tubules compared to using conventional needle irrigation (CI). PIPS technique is based upon photo-acoustic and mechanical action without needing to reach to the root apex. With this technique, each impulse reacts with the water molecules, prompting expansion and serial shock waves that cause the creation of an effective streaming fluid [83]. In a similar manner, the PUI technique is based on the transmission of acoustic energy to an irrigant in the root canal space through ultrasonic waves and can cause acoustic streaming of the irrigant [84]. The higher dentinal tubule penetration for these types of activation may be attributed to the acoustic energy and high-speed fluid motion, regardless of the sealer used.

Aktemur [45] reported that though the removal of the smear layer with a final flush of EDTA and NaOCl did not affect the penetration depth of root canal sealers. Interestingly, the penetration depth of MTA Plus was significantly higher compared to BioRoot RCS when the smear layer was preserved. This could be attributed to the fine particle size and high specific surface area of the powder of this sealer [85]. Lastly, Alim et al. [38] used three different final irrigation solutions (EDTA, MA (maleic acid), and HEBP (etidronic acid)) to evaluate their effect of the sealer penetration. The authors reported that while all the chelation solutions did increase the sealer penetration into the dentinal tubules, using maleic acid (MA) as a final irrigation resulted in a deeper penetration of MTA Flillapex and EndoSequence BC Sealer in the apical region. However, it was acknowledged in that same study that the use of MA or even EDTA solution with NaOCl reduces the desired effects of NaOCl in the irrigation process. This highlights the importance of acknowledging that sealer penetration alone in root canal treatment may not be a crucial factor for the treatment’s success, but a combination of a series of factors.

Mahrani et al. [37] placed the sealer in the root canal with and without ultrasonic activation and found that Endoseal MTA^®^ with sealer ultrasonic activation exhibited a higher sealer penetration percentage than without sealer activation. However, iRoot SP with and without activation showed no significant difference in terms of penetration. According to Akcay el al. [48], this could be attributed to the fact that iRoot SP shows minimal or no shrinkage during setting. It was found in the same study that EndoSeal with ultrasonic activation showed a higher penetration compared to iRoot SP with ultrasonic activation, although it was not significantly different. This may be due to the fact that ultrasonic activation increases flowability and can distribute particles more homogeneously [86,87].

In addition to the methodological differences between the included studies, other factors may also influence dentinal tubule penetration of the sealers, such as root canal morphology. For instance, oval or ribbon-shaped canals occur in approximately 25% of teeth. The preparation and filling of these canals is challenging [88], especially when using the single cone technique. It has been previously reported that despite of effectiveness of a single cone technique in filling of canals prepared by rotary Ni–Ti instruments, its ability to fill an oval or irregular canal space is clearly diminished by its shape [89] and that the filling may result in voids [90,91,92]. Since the recommended filling method with calcium silicate-based based sealers is usually single cone technique, it was suggested to use accessory gutta percha cones for these types of canals to increase the hydraulic force in all directions to push the sealer into the dentinal tubules [47]. Another canal morphology aspect that may influence on sealer penetration is the “butterfly effect”. This is a phenomenon that describes the significantly higher density of dentinal tubules in the buccolingual direction compared with the mesio-distal direction. Interestingly, it produces a characteristic butterfly shape [92]. Teeth with this effect consistently showed a significantly deeper penetration in the bucco-lingual direction compared with teeth without the effect [93]. Therefore, sealer penetration studies should consider the butterfly effect as a potential confounder. Ideally, they should specify whether do the included teeth have this effect, and if so, measurements must be limited to the bucco-lingual direction [93].

It was observed in all the included studies that the dentinal tubule penetration of sealers was the deepest coronally and decreased apically. This can be attributed to various factors, such as the fact that the number and diameter of dentinal tubules decrease toward the apical end of the root canal [94]. Moreover, there is a higher number of sclerosed tubules, sclerotic dentin, and anatomical variation in the apical third than in the coronal third [95]. Another possible reason is the difficulty of extending the endodontic tools to the apical third, making it challenging to apply sufficient irrigation. This may lead to less removal of the smear layer in comparison to the coronal third of the canal. Furthermore, the hindered access to the apical third when using filling devices such as heat carriers may also affect the apical filling efficiency in comparison to the coronal third [95].

Sealer penetration may also depend on their physical properties, such as the rate, which is determined by its consistency and particle size. This has a direct effect on the sealer penetration, since smaller particles sizes may penetrate the dentinal tubules easily. In fact, the small particles of calcium silicate-based sealers (<1 um) represent one of the primary reasons for their higher penetration, even with the SC technique [48]. Sealer chemical properties also may influence their ability to penetrate due their basic pH, which denature the collagen fibers, and their volume expansion of 0.2% with setting [96].

Among the included studies in this systematic review, three parameters were assessed regarding dentinal tubule penetration evaluation: maximum depth of penetration, percentage of sealer penetration and total area of sealer penetration. To do so, a single measurement or several measurements were performed to calculate the deepest penetration, the outlined areas along the canal walls in which sealer penetrated the dentinal tubules were measured, and the distances were divided by the canal circumference to calculate the percentage of sealer penetration. The maximum depth was the mostly measured parameters among the included studies. However, these methods have some limitations. For example, single/multiple measurements may not be representative of the overall penetration. Therefore, it may be advantageous to measure the total dentinal tubule penetration area for better statistical analysis in future studies.

### 4.4. Strengths, Limitations, and Future Perspectives

The strengths of this systematic review reside on the use of standardized methodology to perform the study and report data (PRISMA Statement and PRILE guidelines) and the novelty of the addressed topic. To the author’s knowledge, no systematic review has been performed on this regard. As our main limitation, the methodological heterogeneity among the included studies made it impossible to perform a quantitative synthesis of the data or meta-analysis. The intrinsic in vitro nature of the included studies can also act as a limitation, particularly since the results of the included studies could be overestimated due to the use of Rhodamine B. Therefore, the results should be critically interpreted since they may not fully reflect the behavior of the sealers in the clinical setting, where a series of factors may influence the outcome. Nevertheless, the results from the present review reflect the available evidence on the penetration of calcium silicate-based sealers and may serve as a reference for future studies.

Future studies should assess the tubular penetration of calcium silicate-based sealers in combination with other properties, such as push-out bond strength, bioactivity, and biocompatibility. This would provide a better global picture of their physical, chemical and biological properties. These properties could be compared among calcium silicate-based sealers or with other sealers with calcium in their composition, such as calcium phosphates [97].

The influence of different fluorochromes on the results of dentinal tubule penetration studies should also be further explored, due to the recent controversy among the use of Rhodamine B. An interesting experimental model could be the comparison of dentinal tubule penetration of calcium silicate-based sealers with Rhodamine B and the recently proposed fluorochrome Fluo-3. This would corroborate the adequacy of the fluorochromes for the assessment of tubular penetration.

## 5. Conclusions

Within the limitations of this review and the in vitro nature of the included studies, results indicate that calcium silicate-based sealers exhibit favorable dentinal tubule penetration, which can be affected by the irrigation protocol, sealer activation and the filling method used, and root canal morphology. EndoSequence BC Sealer showed the highest sealer penetration among the tested sealers. The influence of different fluorochromes on the results of dentinal tubule penetration studies should also be further explored. The in vitro nature of the included studies limits the applicability of the results into the clinical setting.

## Figures and Tables

**Table 1:** Search strategy.

Database	Search Strategy	Findings
Scopus	#1 TITLE-ABS-KEY (bioceramic OR silicate)	195,865
	#2 TITLE-ABS-KEY (sealer)	6299
	#3 TITLE-ABS-KEY (endod* OR “root canal”)	89,699
	#4 TITLE-ABS-KEY (penetrat* OR adhesion)	1,089,172
	#1 AND #2 AND #3 AND #4	158
Embase	#1 (bioceramic OR silicate)	27,933
	#2 (sealer)	4092
	#3 (endod* OR “root canal”)	91,791
	#4 (penetrat* OR adhesion)	670,462
	#1 AND #2 AND #3 AND #4	110
PubMed	#1 All fields (bioceramic OR silicate)	53,252
	#2 All fields (sealer)	4287
	#3 All fields (endod* OR “root canal”)	89,526
	#4 All fields (penetrat* OR adhesion)	542,247
	#1 AND #2 AND #3 AND #4	168
WOS	#1 Topic (bioceramic OR silicate)	207,528
	#2 Topic (sealer)	11,993
	#3 Topic (endod* OR “root canal”)	93,644
	#4 Topic (penetrat* OR adhesion)	1,456,666
	#1 AND #2 AND #3 AND #4	225
Cochrane	#1 All text: (bioceramic OR silicate)	452
	#2 All text: (sealer)	578
	#3 All text: (endod* OR “root canal”)	5387
	#4 All text: (penetrat* OR adhesion)	13,938
	#1 AND #2 AND #3 AND #4	19

**Table 2:** Commercially available calcium silicate-based sealers evaluated in the included studies.

Sealer	Manufacturer	Composition	Studies in Which it Was Assessed
**BioRoot RCS**	Septodont, Saint Maur-des-Fosses, France	Powder: tricalcium silicate, zirconium dioxide, and povidone. Liquid: water, calcium chloride, and polycarboxylate [49]	(Muedra et al., 2021) [40], (Marissa et al., 2020) [42], (Kim et al., 2019) [44], (Aktemur Türker et al., 2018) [45] (Arikatla et al., 2018) [46].
**EndoSequence BC Sealer**	Brasseler USA, Savannah, GA, USA	Zirconium oxide, calcium silicates, calcium phosphate monobasic, calcium hydroxide, filler, and thickening agents [50]	(Alim Uysal et al., 2021) [38], (Muedra et al., 2021) [40], (Reynolds et al., 2020) [41], (el Hachem et al., 2019) [43], (McMichael et al., 2016) [47].
**Endoseal MTA**	Maruchi, Wonju, Korea	Calcium silicates, calcium aluminates, calcium aluminoferrite, calcium sulfates, radiopacifier, and thickening agents [50]	(Maharani et al., 2021) [37], (Kim et al., 2019) [44].
**iRoot SP**	Innovative BioCeramix, Vancouver, Canada	zirconium oxide, calcium silicates, calcium phosphate monobasic, calcium hydroxide, filler, thickening agents [51]	(Maharani et al., 2021) [37], (Marissa et al., 2020) [42], (Akcay et al., 2016) [48].
**MTA Fillapex**	Angelus, Londrina, Brazil	Paste A: salicylate resin, bismuth trioxide, fumed silica, Paste B: fumed silica, titanium dioxide, mineral trioxide aggregate, and base resin. [51]	(Alim Uysal et al., 2021) [38], (Marissa et al., 2020) [42], (McMichael et al., 2016) [47], (Akcay et al., 2016) [48].
**NeoMTA Plus**	Avalon Biomed Inc., Bradenton, FL, USA	fine powdered tricalcium and dicalcium silicate, tantalite, calcium sulfate and silica [52]	(McMichael et al., 2016) [47].
**Bio-C Sealer**	Angelus, Londrina, PR, Brazil	Calcium silicates, calcium aluminate, calcium oxide, zirconium oxide, iron oxide, silicon dioxide, dispersing agent [53]	(Eid et al., 2021) [39]
**MTA plus**	Avalon Biomed Inc. Bradenton, FL, USA	Bismuth oxide, portlandite, dicalcium silicate and tricalcium silicate, provided with either water or a gel for mixing [54]	(Aktemur Türker et al., 2018) [45], (Arikatla et al., 2018) [46].
**Endosequence HiFlow**	Brasseler USA, Savannah, GA, USA	Zirconium oxide, calcium silicates, calcium phosphate monobasic, calcium hydroxide, filler, and thickening agents [50]	(Eid et al., 2021) [39], (Reynolds et al., 2020) [41].

**Table 3:** General methodological characteristics.

Author, Year	Sample Size	Sample Storage after Extraction, until Use	Distance from the Tip of K-File to the Apical Foramen	Instrumentation System/Last File Used	Irrigation Sequence
Maharani et al., 2021 [37]	32 premolars	Not reported	1 mm	ProTaper Next (Dentsply Maillefer, Ballaigues, Switzerland) /×4 (40.06)	2.5% NaOCl, 5 mL 17% EDTA.
Alim Uysal et al., 2021 [38]	84 mandibular premolars	Distilled water	1 mm	Protaper Next (Dentsply Maillefer, Ballaigues, Switzerland) /×3 (30.07)	Four groups according to the final irrigation: A. 2 mL of 2.5% NaOCl after each file, with saline as the final irrigation solution. (Control group) B. 2 mL of 2.5% NaOCl after each file, with 17% EDTA for 1 min as the final irrigation solution. C. 2 mL of 2.5% NaOCl after each file, with 7% MA (maleic acid)for 1 min as the final irrigation solution. D. 2 mL of 2.5% NaOCl and 9% HEBP (etidronic acid) after each file, with 2.5% NaOCl and 9% HEBP for 1 min as the final irrigation solution.
Eid et al., 2021 [39]	44 mandibular premolars	Not reported	0.5 mm	ProTaper System (Dentsply Maillefer, Ballaigues, Switzerland)/f3 (30.09)	10 mL 5.25% NaOCl, 10 mL of 17% EDTA, 3 mL of 5.25% NaOCl for 1 min, 10 mL of deionized water as a final flush. irrigants were sonically activated for 1 min using the Endoactivator system (Dentsply Maillefer, Ballaigues, Switzerland) with a 25/04 tip.
Muedra et al., 2021 [40]	60 maxillary and mandibular premolars.	100% humidity environment	1 mm	Mtwo rotary system (VDW, Munich, Germany)/35.04	5.25% NaOCl 17% EDTA during 1 min 5 mL saline
Reynolds et al., 2020 [41]	50 Incisors, canines, and premolars	Formalin	1 mm.	EndoSequence (Brasseler, Sanannah, GA, USA) rotary files/40.06	1 mL of 6% NaOCl prior to instrumentation, 1 mL 6%NaOCl, 3 mL 17% EDTA for 1 min, 3 mL 6% NaOCl for 1 min, 5 mL saline for 1 min.
Marissa et al., 2020 [42]	27 mandibular premolars	0.9% NaCl solution	±0.5 mm	ProTaper Next files (Dentsply, Ballaigues, Switzerland) /×3 (30.07)	17% EDTA gel as a lubricant when changing instruments, 2 mL 2.5% NaOCl and activated with sonic instruments, 2 mL distilled water solution, 17% EDTA for 1 min, 2 mL distilled water.
el Hachem et al., 2019 [43]	96 maxillary central incisors	distilled water	0.5 mm	ProTaper (Dentsply Maillefer, Ballaigues, Switzerland)/F4 (40.06)	10 mL 5.25% NaOCl 10 mL 17% EDTA, 3 mL 5.25% NaOCl for 3 min, 10 mL deionised water.
Kim et al. 2019 [44]	60 premolars	Not reported	0 mm	ProFile rotary instruments (Dentsply Maillefer, Ballaigues, Switzerland)/ 40.06	2 mL of 2.5% NaOCl, 2 mL of 17% EDTA for 1 min, 10 mL of distilled water.
Aktemur Türker et al., 2018 [45]	90 mandibular premolars	Not reported	1 mm	ProTaper Universal file system (Dentsply, Maillefer, Ballaigues, Switzerland)/40.06	2.5% NaOCl during instrumentation specimens were randomly assigned to two groups according to the final irrigation: (n = 45): NaOCl (n = 45) = 3 mL of 17% EDTA for one minute, then 3 mL NaOCl followed by a final flush with 5 mL distilled water.
Arikatla et al., 2018 [46]	60 mandibular premolars	saline, after disinfection with 0.5% chloramine-T solution		ProTaper rotary files (DenTsply Maillefer, Switzerland)/F3 (30.09)	3% NaOCl, 2 mL of 17% EDTA for 1 min, 5 mL of distilled water.
McMichael et al., 2016 [47]	80 bicuspids, canines, and incisors	distilled water Roots were also stored in distilled water at room temperature after instrumentation until filling	1 mm	(EndoSequence, Brasseler USA)/40.06	1 mL NaOCl, 3 mL 17% EDTA for 1 min, 3 mL 6% NaOCl for 1 min, 5 mL saline for 1 min.
Akcay et al., 2016 [48]	156 mandibular premolars	Thymol solution for 48 h for disinfection, then stored in 48 C distilled water.	1 mm	ProTaper Universal rotary instruments (Dentsply, Maillefer)/F4 (40.06)	2 mL of 5% NaOCl 5 mL of 17% EDTA for 1 min 5 mL of 5% NaOCl for 1 min Specimens were randomly subdivided into three groups according to the final irrigation protocol (n=13): CI (Conventional needle irrigation), PIPS (Phophoton-induced-photoacoustic streaming activation), and PUI (passive ultrasonic irrigation).

**Table 4:** Specific study characteristics.

Author, Date	Groups			Dye Used and Mixing Method	Sample Sectioning *	Observation Method	Outcome Measure
	Sealer	Filling Method	n				
Maharani et al., 2021 [37]	iRoot SP without Ultrasonic activation	SC	8	0.1% rhodamine B dye (Sigma-Aldrich, St. Louis, MO, USA)	5	CLSM	Mean maximum sealer penetration depth (μm)
	iRoot SP with Ultrasonic activation	SC	8				
	Endoseal MTA Without ultrasonic activation	SC	8				
	Endoseal MTA with Ultrasonic activation	SC	8				
Alim Uysal et al., 2021 [38]	MTA Fillapex	SC	28	0.01% rhodamine B (Bereket Kimya, Istanbul, Turkey)	2, 6 and 10	Cytation 5 Cell Imaging Multimode Reader and Gen5 software	Maximum sealer penetration depths (μm). mean sealer penetration depths (μm).
	EndoSequence BC Sealer	SC	28				
Eid et al., 2021 [39]	Bio-C sealer	SC	10	0.1% Rhodamine B dye (Sigma-Aldrich, St. Louis, MO, USA)	1 and 5	CLSM.	Mean maximum sealer penetration depth (μm).
	Bio-C sealer	WVC	10				
	HiFlow	SC	10				
	HiFlow	WVC	10				
	Control group—filled with either HiFlow sealer or Bio-C without the fluorescent agent		2				
	Control group— Not obturated		2				
Muedra et al., 2021 [40]	EndoSequence BC Sealer	SC	20	0.1% Rhodamine B, C28H31ClN2O3, (Panreac Químicas S.A.U. Casteller del Vallès, BCN, Spain).	3, 5, and 8 (Samples were stored in a light-free environment to avoid a previous exposure of the fluorochrome to light before it was viewed under CLSM.)	CLSM	Median Tubular Penetration Depth. Median Percentage of Perimeter Penetrated.
	BioRoot RCS	SC	20				
	Control Group: AH Plus	SC	20				
Reynolds et al., 2020 [41]	Control group: 2Seal easymiX,	WVC	10	rhodamine B fluorescent dye (Sigma-Aldrich, St. Louis, MO, USA)	3 and 6	CLSM To evaluate the maximum depth of sealer penetration, the distance between the deepest point of sealer penetration to the root canal wall was measured using imageJ software.	Median maximum sealer penetration depth. Median percentage of sealer penetration.
	EndoSequence BC	SC	10				
	EndoSequence BC	WVC	10				
	EndoSequence BC hiflow	SC	10				
	EndoSequence BC hiflow	WVC	10				
Marissa et al., 2020 [42]	IRoot^®^ SP	SC	9		5	SEM	Mean maximum sealer penetration depth (μm)
	MTA^®^ Fillapex	SC	9				
	BioRoot™ RCS	SC	9				
el Hachem et al., 2019 [43]	EndoSequence BC Sealer	SC	32	0.1% rhodamine B (Sigma-Aldrich, St. Louis, MO, USA)	1 and 5	CLSM	Maximum sealer penetration depth (μm). Mean sealer penetration depth (μm).
	new experimental novel tricalcium silicate (NTS)-based sealer	SC	32				
Kim et al., 2019 [44]	BioRoot RCS	SC	20	0.1% rhodamine B dye (Sigma-Aldrich, St. Louis, MO, USA)	3, 5 and7	CLSM	maximum sealer penetration depth (μm). mean fluorescence intensity. sum fluorescence intensity
	Endoseal MTA	SC	20				
Aktemur Türker et al., 2018 [45]	BioRoot RCS	SC	30	0.1% rhodamine B dye (Sigma Aldrich Co., St Louis, MO, USA)	Mid third	CLSM	Mean of Push-out bond strength values. Sealer mean penetration depth (mm) Penetration mean percentage %
	MTA Plus	SC	30				
Arikatla et al., 2018 [46]	MTA plus	LC	10	isothiocyanate fluorescent 0.1% Rhodamine dye (Macsen Labs Pvt Ltd., Rajasthan)	3 and 6	CLSM.	sealer mean penetration depth (μm). dentin sealer interfacial gaps (μm).
	BioRoot RCS	LC	10				
McMichael et al., 2016 [47]	EndoSequence BC sealer	SC	10	Rhodamine dye (n/s)	1 and 5	CLSM	Maximum sealer penetration depth (μm). Percentage of sealer penetration %
		WVC	10				
	MTA Fillapex	SC	10				
		WVC	10				
	NeoMTA Plus	SC	10				
		WVC	10				
	QuickSet2	SC	10				
		WVC	10				
Akcay et al., 2016 [48]	iRoot SP	SC	39	0.1% fluorescent rhodamine B isothiocyanate (n/s)	2, 5, and 8	CLSM	Mean total dentinal tubule penetration area values (mm^2^).
	MTA Fillapex	SC	39				

SC: Single cone. WVC: Warm vertical compaction. n/s: not specified. * Distances from the apex in mm. SEM: scanning electron microscopy. CLSM: confocal laser scanning microscopy.

**Table 5:** Qualitative significant sealer penetration Depth results.

Author, Year	Distance from Apex (mm)	Sealer Penetration Depth	*p* Value
Alim Uysal et al., 2021 [38]	2 mm	Maleic acid for final irrigation > EDTA and HEBP for final irrigation (in all sealers: MTA Fillapex, EndoSequence BC)	0.013
	6 mm		
	10 mm		
Eid et al., 2021 [39]	1 mm	(endosequence BC HiFlow, Bio-C sealer): WVC > SCO	0.011
	5 mm	(endosequence BC HiFlow, Bio-C sealer): WVC > SCO	0.034
Muedra et al., 2021 [40]	3 mm		
	5 mm	EndoSequence > BioRoot RCS	<0.05
	8 mm	EndoSequence > BioRoot RCS	<0.05
Marissa et al., 2020 [42]	5 mm	MTA Fillapex ˃ IRoot^®^ SP ˃ BioRoot™ RCS	<0.001
Kim et al., 2019 [44]	3 mm		
	5 mm	BioRoot RCS > Endoseal MTA.	<0.017
	7 mm	BioRoot RCS > Endoseal MTA.	<0.017
Aktemur Türker et al., 2018 [45]	Mid third	Smear layer preserved: MTA Plus > BioRoot RCS. Smear layer removed: MTA Plus > BioRoot RCS.	<0.05
McMichael et al., 2016 [47]	1 mm	MTA Fillapex with WVC technique > MTA Fillapex with SC technique. EndoSequence with WVC technique > EndoSequence with SC technique.	<0.0125
	5 mm	Endodecuence Sealer, MTA Fillapex > QuickSet2 (when used with the WVC technique compared with the SC technique).	<0.0125

SC: Single cone. WVC: Warm vertical compaction.

## Data Availability

Additional data can be obtained upon reasonable request from the corresponding author.

## Supplementary Materials

The following supporting information can be downloaded at: https://www.mdpi.com/article/10.3390/ma16072734/s1, Supplementary Table S1: Mean ± (standard deviation or standard error*) results of tubular penetration depth & percentage. Supplementary Table S2: Median (M), Min, Max and Interquartile range (IQR) results of tubular penetration depth and percentage. Supplementary Table S3: Quality assessment of the included studies.
