# Supplementary material for: Calcium Silicate-Based Sealer Dentinal Tubule Penetration—A Systematic Review of In Vitro Studies
Source: Materials (Basel). 2023 Mar 29;16(7):2734. doi: 10.3390/ma16072734 (PMC10096084; doi:10.3390/ma16072734)
Supplement: Supplementary file 1 [file materials-16-02734-s001.zip › materials-2256784-supplementary.pdf]

Supplementary table 1. mean ± (standard deviation or standard error*) results of tubular penetration depth & percentage.

Author/ date	Sealer, filling method	Tubular penetration distance/ Depth (um)				Percentage of sealer penetration %			
		apical	middle	coronal	total	apical	middle	coronal	total
(Maharani et al., 2021) [37]	iRoot SP without SUA, SC				638.13 (166.45)				
	iRoot SP with SUA, SC				651.50 (236.63)				
	Endoseal MTA Without SUA, SC				580.13 (121.25)				
	Endoseal MTA with SUA, SC				914.25 (231.84)				
(Alim Uysal et al., 2021) [38]	AH plus, SC	444,40 (142,09)	705,65 (107,96)	977,35 (228,67)					
	MTA Fillapex, SC	457,60 (141,04)	726,50 (111,66)	1,007,55 (196,64)					
	Endosequence BC sealer, SC	469,80 (93,39)	848,50 (140,95)	1,092,05 (198,23)					
Eid et al., 2021) [39]	Bio-C sealer, SC	397.428 ± 77.46	1080.92 ± 575.228						
	Bio-C sealer, WVC	447.076± 303.082	1421.98±509.75						
	HiFlow, SC	194.24 ± 227.369	1115.051 ± 619.506						
	HiFlow, WVC	672.82 ± 390.807	1567.634 ± 666.873						
(Marissa et al., 2020) [42]	IRoot® SP, SC		115.99 (13.1)						
	MTA® Fillapex, SC		209.28 (34.31)						
	BioRoot™ RCS, SC		84.07 (5.6)						
(el Hachem et al., 2019) [43]	AH Plus	568.96 ± 222.89	984.29 ± 271.55						
	EndoSequence BC	600.84 ± 235.93	1152.81 ± 257.51						
	NTS	656.89 ± 272.93	1063.61 ± 293.52						
(Aktemur Türker et al., 2018) [45]	AH 26, SC, With smear layer		1413 ± 330 *				73.00± 20.2		
	BioRoot RCS, SC, With smear layer		1248 ± 390 *				75.83± 27.1		
	MTA Plus, SC, With smear layer		1972 ± 330 *				75.67± 19.5		
	AH 26, SC, No smaer layer		1443 ± 100 *				78.50 ± 17.4		
	BioRoot RCS, SC,		999 ± 360 *				68.33 ± 28.0		

	No smaer layer								
	MTA Plus, SC,<br>No smaer layer		1701 ± 390 *				88.08 ± 12.5		
(Arikatla et al., 2018) [46]	AH plus, LC				309.55 (113.82)				
	MTA plus, LC				192.54 (55.16)				
	BioRoot RCS, LC				221.00 (59.37)				

SUA: Sealer Ultrasonic activation, **SC**: Single cone, **WVC**: Warm vertical compaction, **LC**: Lateral condensing, *****: distance in millimetres (mm).

Supplementary table 2. Median (M), Min, Max and Interquartile range (IQR) results of tubular penetration depth and percentage.

Author/ Date	Sealer	Tubular penetration distance / depth (um)												percentage of sealer penetration %											
		Apical				Middle				Coronal				Apical				Middle				Coronal			
		M	min	max	IQR	M	min	max	IQR	M	min	max	IQR	M	min	max	IQR	M	min	max	IQR	M	min	max	IQR
(Muedra et al., 2021) [40]	EndoSequence BC Sealer, SC	475.35			404.81	981.33			581.14	1316.57			804.57	33.65			52.15	49.73			44.23	55.64			43.23
	BioRoot RCS, SC	304.32			505.02	508.11			715.84	743.74			781.18	9.76			29.75	24.08			41.7	28.41			34.98
	(Control), AH Plus, SC	193.25			431.55	449.03			612.64	1118.01			727.53	11.8			13.25	39.67			28.83	60.37			27.14
(Reynolds et al., 2020) [41]	2Seal easymiX, WVC	0.9 *	0.2 *	1.8 *						1.6 *	0.8 *	2.6 *		79.5	49	100						100	49	100	
	EndoSequence BC, SC	0.9 *	0.1 *	1.8 *						2.1 *	0.3 *	2.3 *		79.5	4	100						100	45	100	
	EndoSequence BC, WVC	0.9 *	0.4 *	1.8 *						1.9 *	0.8 *	2.3 *		93.5	61	100						100	91	100	
	EndoSequence BC hiflow, SC	1 *	0.5 *	1.9 *						2.1 *	1.3 *	2.3 *		80	49	100						100	69	100	
	EndoSequence BC hiflow, WVC	1.1 *	0.6 *	1.8 *						1.8 *	1.1 *	2.5 *		77.5	53	100						100	80	100	

SC: Single cone, **WVC**: Warm vertical compaction, *****: distance in millimetres (mm).

Supplementary table 3. Quality assessment of the included studies.

	**1**		**2**	**3**					**4**		**5**										**6**			**7**					**8**		**9**	**10**	**11**								
	a	b	a	a	b	c	d	e	a	b	a	b	c	d	e	f	g	h	i	j	a	b	c	a	b	c	d	e	a	b	a	a	a	b	c	d	e	f	g	h	**%**
(Maharani, Ricardo, Ayu, et al., 2021) [37]	N	Y	Y	N	Y	N	Y	Y	Y	Y	Y	N	Y	Y	Y	NA	NA	NA	NA	N	Y	N	Y	Y	Y	Y	N	N	Y	Y	Y	Y	Y	Y	Y	Y	Y	Y	Y	Y	78
(Alim Uysal et al., 2021) [38]	N	Y	Y	N	Y	Y	Y	Y	Y	Y	Y	N	N	Y	Y	NA	NA	NA	NA	Y	N	N	Y	Y	Y	Y	Y	Y	Y	Y	Y	Y	Y	Y	Y	Y	Y	Y	Y	Y	83
(Eid et al., 2021) [39]	Y	Y	Y	N	Y	Y	Y	Y	Y	Y	Y	N	Y	Y	Y	NA	NA	NA	NA	Y	Y	N	Y	Y	Y	N	N	Y	Y	Y	N	Y	Y	Y	Y	Y	Y	Y	Y	Y	83
(Muedra et al., 2021) [40]	Y	Y	Y	Y	Y	Y	Y	Y	Y	Y	Y	Y	Y	Y	Y	NA	NA	NA	NA	Y	Y	N	Y	Y	Y	Y	Y	Y	Y	Y	Y	Y	Y	Y	N	Y	Y	Y	Y	Y	94
(Reynolds et al., 2020) [41]	Y	Y	Y	N	Y	N	Y	Y	Y	Y	Y	N	Y	Y	Y	NA	NA	NA	NA	N	N	Y	N	Y	Y	N	Y	Y	Y	Y	Y	Y	Y	Y	Y	Y	Y	Y	Y	Y	81
(Marissa et al., 2020) [42]	N	Y	Y	Y	Y	N	Y	Y	Y	Y	N	N	N	Y	Y	NA	NA	NA	NA	Y	Y	N	Y	Y	Y	N	N	N	Y	Y	Y	N	Y	Y	N	Y	Y	Y	Y	Y	69
(el Hachem et al., 2019) [43]	Y	Y	Y	N	Y	N	Y	Y	Y	Y	Y	N	N	Y	Y	NA	NA	NA	NA	Y	Y	N	Y	Y	Y	N	Y	Y	Y	Y	Y	Y	Y	Y	Y	Y	Y	Y	N	Y	81
(Kim et al., 2019) [44]	Y	Y	Y	N	Y	Y	Y	Y	Y	Y	Y	Y	N	Y	Y	NA	NA	NA	NA	N	N	N	Y	Y	Y	Y	N	N	Y	Y	Y	Y	Y	Y	Y	Y	Y	Y	Y	Y	81
(Aktemur Türker et al., 2018) [45]	N	Y	Y	N	Y	N	N	Y	Y	Y	N	N	N	Y	Y	NA	NA	NA	NA	N	Y	N	Y	Y	Y	N	N	N	Y	Y	Y	Y	Y	Y	N	N	Y	Y	Y	Y	61

(Arikatla et al., 2018) [46]	N	Y	Y	Y	Y	Y	Y	Y	Y	Y	N	N	N	Y	Y	NA	NA	NA	NA	Y	Y	N	Y	Y	Y	N	N	Y	Y	Y	Y	Y	Y	Y	N	Y	Y	Y	N	Y	75
(McMichael et al., 2016) [47]	N	Y	Y	Y	Y	N	Y	Y	Y	Y	Y	N	N	Y	Y	NA	NA	NA	NA	N	N	N	Y	Y	Y	N	N	N	Y	Y	Y	Y	Y	Y	Y	Y	Y	Y	N	Y	69
(Akcay et al., 2016) [48]	Y	Y	Y	Y	Y	Y	Y	Y	Y	Y	N	N	N	Y	Y	NA	NA	NA	NA	Y	Y	N	Y	Y	Y	N	Y	Y	Y	Y	Y	Y	Y	Y	N	N	Y	Y	N	Y	78
																																									**78**

Quality assessment of included studies using the PRILE 2021 guidelines for reporting laboratory studies in Endodontology [34].

Y: item reported in the study, **N**: item not reported in the study, **NA**: Non-applicable.
